# Is exposure in vivo cost-effective for chronic low back pain? A trial-based economic evaluation

**DOI:** 10.1186/s12913-015-1212-6

**Published:** 2015-12-14

**Authors:** Marielle E. J. B. Goossens, Reina J. A. de Kinderen, Maaike Leeuw, Jeroen R. de Jong, Joop Ruijgrok, Silvia M. A. A. Evers, Johan W. S. Vlaeyen

**Affiliations:** Department of Clinical Psychological Science, Maastricht University, P.O. Box 616, 6200 MD Maastricht, The Netherlands; CAPHRI - School for Public Health and Primary Care, Department of Rehabilitation Research, Maastricht University, P.O. Box 616, 6200 MD Maastricht, The Netherlands; CAPHRI - School for Public Health and Primary Care, Department of Health Services Research, Maastricht University, P.O. Box 616, 6200 MD Maastricht, The Netherlands; Adelante, Rehabilitation Foundation Hoensbroek, Zandbergseweg 111, 6432 CC, Hoensbroek, The Netherlands; Department of Rehabilitation, Maastricht University Medical Center, Maastricht, The Netherlands; Trimbos Institute, Netherlands Institute of Mental Health and Addiction, P.O. Box 725, 3500 AS Utrecht, The Netherlands; Health Psychology Research Group, University of Leuven, Tiensestraat 102, 3000 Leuven, Belgium

**Keywords:** Chronic low back pain, exposure in vivo, economic evaluation, costs, QALYs

## Abstract

**Background:**

Back pain is one of the most expensive health complaints. Comparing the economic aspects of back pain interventions may therefore contribute to a more efficient use of available resources. This study reports on a long-term cost-effectiveness analysis (CEA) and cost-utility analysis (CUA) of two treatments as viewed from a societal perspective: 1) exposure in vivo treatment (EXP), a recently developed cognitive behavioral treatment for patients with chronic low back pain who have elevated pain-related fear and 2) the more commonly used graded activity (GA) treatment.

**Methods:**

Sixty-two patients with non-specific chronic low back pain received either EXP or GA. Primary data were collected at four participating treatment centers in the Netherlands. Primary outcomes were self-reported disability (for the CEA) and quality-adjusted life years (for the CUA). Program costs, health care utilization, patient and family costs, and production losses were measured by analyzing therapy records and cost diaries. Data was gathered before, during, and after treatment, and at 6 and 12 months after treatment. Non-parametric bootstrap analyses were used to quantify the uncertainty concerning the cost-effectiveness ratio. In addition, cost-effectiveness planes and cost-effectiveness acceptability curves were performed.

**Results:**

EXP showed a tendency to reduce disability, increase quality adjusted life years and decrease costs compared to GA. The incremental cost-effectiveness ratios of both the CEA and CUA are in favor of EXP.

**Conclusions:**

Based on these results, implementing EXP for this group of patients seems to be the best decision.

**Trial registration:**

ISRCTN88087718

## Background

Non-specific chronic low back pain (CLBP) is not only one of the most common health problems in Western societies, it also is one of the most expensive in terms of health care costs and costs to society [1]. The economic consequences of chronic pain are felt not only by the patient; other parties, such as the family, social environment, health care providers, and employers, are all directly or indirectly affected by the consequences of CLBP. In the Netherlands, the total costs of back and neck pain have been estimated at 1–2 % of the gross national product [2, 3]. In recent decades, ‘disorders of the musculoskeletal system’ and ‘symptoms and ill-defined conditions’, which include non-specific CLBP, were among the top five most expensive disorders in the Netherlands [4, 5]. These figures are comparable to those from Australia, Canada, France, Germany, Japan, Spain, the United Kingdom, and the United States [1]. Overall, costs arising from productivity loss account for more than 50 % of the total costs associated with CLBP [6–9].

The most favorable treatment approach for non-specific CLBP incorporates cognitive behavioral therapy (CBT) techniques. Meta-analyses have concluded that CBT treatments have positive effects on pain, disability, and mood, both post-treatment and at follow-up [10]. Theory-based therapies are also vital to increase the benefits for CLBP patients and decrease costs [11]. In recent years, evidence has accumulated to show that catastrophic beliefs about pain, pain-related fear and associated avoidance behaviors are instrumental in the development and maintenance of CLBP [12, 13]. In common CBT approaches, such as graded activity, increasing activity levels is promoted irrespective of the degree of fear. Fearful patients may benefit from these interventions.

Exposure in vivo (EXP) is a treatment that was specially developed to reduce excessive pain-related fear. By systematically exposing patients to painful movements and activities that they have been avoiding, an opportunity is created to correct their catastrophic misinterpretations of the association between movements and harm. So far, EXP treatment has proven to be effective in improving functional disability by diminishing pain catastrophizing and pain-related fear [14–17].

EXP treatment is different from the more standard graded activity (GA) treatment [18]. Whereas GA focuses on positively reinforcing healthy behaviors and activity levels, EXP specifically targets the reduction of pain-related fear. In a multicenter randomized controlled trial, Leeuw et al. [19] reported that EXP was superior to GA for reducing the perceived harmfulness of daily activities in CLBP patients. The treatments did not differ significantly for functional disability, though the difference almost reached significance in favor of EXP.

Information on effectiveness alone is not enough to draw definite conclusions about the implementation of either intervention. For example, when the effectiveness of interventions is equal or did not differ significantly, one intervention could result in other benefits, such as reduced health care costs, reduced patient and family related costs, and/or reduced productivity losses. Since resources are scarce, current health care decision-making is necessarily evidence-based, requiring proof that the effects of new interventions are actually worth the extra cost of the intervention. Additional information is therefore needed about the incremental costs of EXP versus GA. Given the wide societal impact of the economic consequences, it can be expected that, in this group of patients, EXP will lead to fewer health care costs, patient and family-related costs, and indirect costs.

The aim of this study therefore was to examine the cost-effectiveness (CEA) and the cost-utility (CUA) of EXP compared to GA from a societal perspective, using CLBP patients from the study conducted by Leeuw et al. [19]. We hypothesized that although the effectiveness of EXP and GA treatment may differ only slightly or not at all, there would be a difference in utilities and societal costs in favor of EXP, thus recommending implementation of EXP.

## Methods

### Design and patients

In a randomized controlled multicenter trial conducted by Leeuw et al. [19], 85 CLBP patients were randomly assigned to EXP (42 patients) or GA (43 patients) treatment. Patients either were referred by physicians at various outpatient facilities and hospital departments in the southern part of the Netherlands or responded to an advertisement in a local newspaper. Patients were included if they were between the ages of 18 and 65 and if their lower back pain had lasted for at least three months. Furthermore, patients had to be sufficiently disabled (Roland Disability Score >3) and at least moderately fearful of movement, injury or reinjury (Tampa Scale of Kinesiophobia >33). Patients were excluded if they were illiterate, pregnant, involved in any litigation concerning their ability to work or disability income, or engaged in substance abuse that could interfere with treatment. Patients with specific medical disorders, cardiovascular diseases, or serious psychopathology were also excluded.

### Ethics

The Medical Ethics Committee of Maastricht University Medical Centre + approved the research protocol, as did the institutional committees at the participating institutions. Participation in the study was on the basis of informed consent.

### Randomization and design

Patients were randomized to either EXP or GA treatment at one of the four participating treatment centers following a predetermined and computer-generated randomization schedule, pre-stratified by therapist team and the degree of pain catastrophizing and disability. For disability and pain catastrophizing, the median score of the data from a previous randomized controlled trial was used as the cut-off [20]. Within each stratum, we used a randomized block design with a block size of two. The randomization schedule was only accessible to the research assistant performing the randomization and concealed for those involved in patient recruitment. The randomization was also concealed for the therapists until the time when treatment started. Since there was one team for both treatments the therapists were not blinded to the content of the treatments being compared.

Treatment assignment was concealed from participants until they arrived at their first intervention session. After the second pre-treatment measurement, patients received a sealed envelope from the research assistant containing a sheet of colored paper indicating their treatment assignment, which they opened together with the psychologist during the intake.

The data analysts (ML, MG, RdK) were blinded during all the data analyses. Blinding (patient allocation) was broken afterwards.

### Interventions

#### Graded exposure in vivo

EXP aims improve functional ability by systematically reducing pain-related fear. The assumption is that patients have developed propositional knowledge about the association between movements and aversive outcomes, as a result of which protective responses such as avoidance and escape behavior occur [21]. During exposure to the conditioned stimuli, patients may experience that they actually overestimated the threat, correct the catastrophic expectations and learn to inhibit their avoidance response [22, 23]. The program consisted of 16 one-hour sessions and was provided according to a standardized protocol.

EXP treatment started by analyzing a patient’s catastrophic misinterpretations and using the Photograph Series of Daily Activities (PHODA) to establish a personally tailored, graded hierarchy of fear-eliciting activities [24]. This was followed by two educational sessions led by the rehabilitation physician and the therapist mini-team (consisting of a psychologist and a physical or occupational therapist). During these sessions, they explained the treatment rationale to the patient by integrating the individual complaints and the patient’s characteristics in the fear-avoidance model. They further explained that protective behaviors may have been adaptive during the acute pain episode, but that they paradoxically worsen the pain problem in the chronic phase. During the remaining sessions, patients were gradually exposed to the fear-provoking activities and/or movements according to the fear hierarchy established by means of PHODA. A more detailed description of EXP can be found elsewhere [17, 19].

#### Graded activity

GA was developed by Wilbert Fordyce in the early 1970s and aims to improve functional ability by positively reinforcing healthy behaviors and activity levels using operant learning principles [25]. Here, the GA treatment consisted of approximately 25 one-hour sessions provided according to a standardized protocol. GA started with a one-session psychological intake followed by two educational sessions (led by the rehabilitation physician and the therapist mini-team, respectively) in which the treatment rationale was explained, emphasizing the negative effects of inactivity and the positive effects of physical activity on well-being. Individual treatment goals and baseline activity level were then identified. In the subsequent sessions, a time-contingent treatment schedule was initiated in which the amount of activity was gradually increased using an individualized quota system [25]. In contrast to EXP, the psychologist’s role in GA was limited to two sessions at which participants’ family members were helped to positively reinforce any progress made during the treatment. More information about GA is available elsewhere [19, 26].

### Data collection

Before treatment, data was gathered about gender, age, education level, employment status, and pain duration. Additionally, fear of movement/reinjury was assessed using the Tampa Scale for Kinesiophobia (TSK: [27]).

### Cost measures

To evaluate the economic consequences of treatment from a societal perspective, we assessed the intervention costs, other health care costs, patient and family costs, and productivity losses. The intervention costs (GA = €1,969.39 and EXP = €2,166.84) have been calculated on the basis of the time that the rehabilitation physician, psychologist, and physical/occupational therapist spent on the therapy. Travel expenses to the intervention have been calculated based on the mean distance multiplied by standard cost prices [28]. Other health care costs concern all other pain-related health care utilization, including visits to the general practitioner, specialist care, alternative health care, consultations with a physical or occupational therapist, hospitalization, and prescribed or over-the-counter medication. Patient and family costs include out-of-pocket costs, such as costs for home care, informal care, and additional expenses. Productivity losses have been calculated based on days absent from work due to back pain.

Patients maintained a monthly cost diary that was used to measure all health care costs, patient and family costs, and productivity losses [29]. The time horizon of the economic evaluation was 15 months from the start of the treatment to the last follow-up measurement. Each cost diary was filled out two months before treatment (to study baseline differences), during the three months of treatment, and at months 1 and 2, 5 and 6, and 11 and 12 after treatment. Patients were included in the economic analysis if cost diary information was available for at least three months after treatment.

The total costs were estimated using a bottom-up approach, where information on each element of service used was multiplied by an appropriate standardized unit cost and added up to arrive at an overall total cost [30]. For the cost valuation, we used standardized cost prices from the Dutch manual for cost analysis in health care research [28]. Where no standardized cost prices were available, real tariffs or costs were used. Costs of medication were calculated using guideline prices [31]. Additional prescription charges for prescribed medication were also taken into account. Where medication data were varied, the lowest cost price for the specific medication was used. We calculated prices of informal care using standardized cost prices based on general hourly wages. Additional expenses were determined using the costs patients recorded in their cost diaries.

Productivity losses were calculated according to the Human Capital Approach, which estimates the value of the potential production loss over an entire period of absenteeism. Finally, productivity costs were calculated by multiplying the number of days of absence from work by the cost per day. Another method to measure productivity losses is the Friction Cost Method, which is based on the assumption that an organization needs a certain time span (friction period) to replace an absent worker with another worker. The definitive number of days of work absence is then limited to the duration of the friction period, which has been determined to be 23 weeks in the Netherlands [28]. To deal with the methodological uncertainty of choosing the Human Capital Approach, productivity costs were also estimated using the Friction Cost Method in a sensitivity analysis. Costs were expressed in euros using the 2014 price level; if necessary, prices were indexed using rates from Statistics Netherlands.

### Outcome measures

Functional disability was assessed using the Quebec Back Pain Disability Scale (QBPDS) [32, 33]. Patients were asked to rate their difficulty in performing 20 activities commonly affected by back pain on a seven-point rating scale ranging from 0 (no trouble) to 6 (unable to). Total scores ranged between 0 and 100. The QBPDS has high internal consistency, good test-retest reliability and sufficient responsiveness and validity [34, 35].

Utilities were based on the Short Form-36 (SF-36). The SF-36 is a reliable and valid instrument for measuring health-related quality of life [36]. Utilities are values or preferences that respondents assign to a particular health state and are expressed overall on a scale from 0 to 1 [30]. The utilities used in this study were derived using an algorithm of the SF-6D, which estimates utilities based on the health-related quality of life scores from the SF-36 [37]. In this procedure, respondents were initially asked to answer questions on the eight subscales of the SF-36 (physical functioning, social functioning, limitations in physical functioning, limitations in emotional functioning, mental health, bodily pain, vitality, general health). The derived utilities at the four measurement points were used to compute the quality-adjusted life year (QALY) score by using the area under the curve method [38]. This means that the utilities were multiplied by the time in that particular health state and then added up to calculate the total number of QALYs.

Outcome measures were assessed twice at baseline (pre-treatment 1 and pre-treatment 2 two weeks later), during treatment, directly after termination of treatment (post-treatment), and at 6 and 12 months after treatment.

### Statistical analyses

The primary analyses were performed according to the intention-to-treat principle, meaning that we used data from all participants, regardless of whether they received the intervention. The analysis included patients for whom at least three months of post-baseline data were registered in the diaries. The costs per patient were calculated by extrapolating the observed costs of each patient to a 15-month period (treatment period and follow-up). Since our time horizon only just exceeded a period of one year, we decided not to discount for these three months. Missing data for outcomes were replaced by the last value carried forward method.

To verify whether randomization resulted in equal treatment groups, we tested the pre-treatment scores of the QBPDS, utilities, QALYs, and pre-treatment costs through independent T-testing or non-parametric bootstrapping. Distribution normality was checked by investigating skewness and kurtosis. Normally distributed variables were tested using independent T-testing and non-normally distributed variables were tested using non-parametric bootstrapping (1,000 replications). The non-parametric bootstrap method estimates sampling distribution by taking repeated samples with replacement from the original participant data [39]. Despite the usual skewness in the distribution of costs, arithmetic means are generally considered to be the most appropriate measure for describing cost data [40].

Additionally, bootstrapping was used to explore sample uncertainty (5,000 replications). These bootstrap simulations were conducted to quantify uncertainty concerning the incremental cost-effectiveness ratio (ICER), yielding information about the joint distribution of cost and effect differences between EXP and GA.

Treatment choice depends on the maximum amount of money that society is prepared to pay for a gain in effectiveness, which is a ceiling ratio. Currently, the Dutch Council for Public Health and Health Care recommends that only treatments resulting in €80,000 per QALY or less should be eligible for reimbursement in diseases with the highest burden, down to €16,000 per QALY in case of the lowest disease burden [41]. As there is much uncertainty surrounding this ceiling ratio, the results of this study are also presented in a cost-effectiveness acceptability curve (CEAC). The CEAC represents the uncertainty concerning the cost-effectiveness of EXP compared to GA treatment by looking at a range of ceiling ratio values [42].

In addition, the sensitivity of the research was measured using one-way sensitivity analysis, which explores how changing the value of one parameter while leaving the others unchanged impacts the results [30]. We used this to analyze the treatments’ impacts on cost-effectiveness when the Friction Costs Method is used instead of the Human Capital Approach to calculate productivity costs. Statistical analyses were performed using SPSS statistical software version 20. Bootstrapping simulations were run in Microsoft Excel.

## Results

### Patients and comparability

After screening, 85 patients were included in the study: 42 were randomized to EXP treatment and 43 to GA treatment. An extensive flowchart of patient data, including withdrawals and reasons for dropout and non-response before and during the intervention, has been published by Leeuw et al. [19]. Overall reasons for non-response and dropout were unrelated to the content of the treatment. Sixty-two patients completed enough diary entries during the months of treatment and follow-up to be included in the economic analysis: 72.1 % in the GA group (31 out of 43 patients) and 73.8 % in the EXP group (31 out of 42 patients). Baseline characteristics (age, gender, education, pain duration, disability, costs) of patients excluded from the economic evaluation were not significantly different from those of the 62 patients included (all *p* > .10; data not presented).

There were no statistical differences between the GA and EXP groups as regards demographic variables, back pain complaints, education level, work status, or level of fear of movement (all *p* > .10) at baseline. The mean age of the 62 participants was 46.3 years (SD = 8.98); 50 % were women, 37 % were employed, 24 % were on sick leave and 27 % received disability pension. They had an intermediate (48.4 %) or low level of education (43.5 %). Their mean level of pain-related fear was 42.1 (SD = 6.28). Participants reported having pain for on average 11.9 years (SD = 10.22). There was also no difference in costs, utilities, or disability before the start of the interventions (see Table 1).

**Table 1:** Mean (SD) and differences in demographic characteristics, pain-related fear, outcome variables, and cost components at an eight-week baseline period per treatment group

	Mean (SD)		Mean difference (95 % CI)	
Variables	GA (*n* = 31)	EXP (*n* = 31)	EXP versus GA	
Age (years)	45.45 (8.42)	47.13 (9.58)	−1.67	(-2.90 to 6.26)
Gender (% female)	50 %	50 %		
Education (%)				(-0.51 to 0.13)
Low	41.9 %	45.2 %		
Intermediate	41.9 %	54.8 %		
High	16.1 %	0 %		
QBPDS (0-100)^b^	53.53 (12.79)	55.02 (11.41)	1.48	(-4.67 to 7.64)
Utility (0-1)^b^	0.60 (0.07)	0.58 (0.05)	−0.02	(-0.06 to 0.01)
Health care costs^a^	68.56 (30.27)	65.78 (38.68)	−3.00	(-94.00 to 99.00)
Patient and family costs^a^	332.44 (94.59)	367.74 (84.98)	35	(-224.00 to 268.00)
Productivity loss^a^	1,126.98 (355.07)	754.83 (255.86)	−372	(-1,205.00 to 456.00)
*Total costs* ^a^	*1,529.32 (371.80)*	*1,204.73 (282.12)*	*−325*	*(-1,301 to 586)*

*GA* graded activity, *EXP* gradual exposure, *QBPDS* Quebec Back Pain Disability Score, *QALYs* quality-adjusted life years. Costs are in euros and per patient

^a^Difference in mean (95 % confidence interval) derived using bootstrapping (1,000 replications)

^b^Difference in mean (95 % confidence interval) derived using independent t-tests or chi-square tests

### Outcomes

Table 2 shows the change in disability and utilities (from the start of treatment through 15-month follow-up) and QALYs gained during the follow-up period. Although EXP patients showed a larger improvement on disability and generic quality of life compared to GA patients, the difference did not reach statistical significance (*p* > .30).

**Table 2:** Mean scores at 15-month follow-up for mean outcome variables and total costs (in euros) per patient per treatment group

	Mean (SD)		Mean difference (95 % CI)
	GA (*n* = 31)	EXP (*n* = 31)	EXP versus GA
QBPDS^b^	40.42 (22.34)	38.19 (20.84)	−2.23 (-13.20 to 8.75)
Utility (SF36)^b^	0.68 (0.14)	0.66 (0.14)	−0.15 (-0.08 to 0.05)
QALY^b^	0.82 (0.12)	0.83 (0.13)	0.01 (-0.6 to 0.07)
Treatment costs	1,969.39 (0)	2,166.84 (0)	197.45
Health care costs^a^	1,841.72 (595.12)	1,011.67 (220.68)	−830 (-2,209 to 247)
P & F costs^a^	5,347.25 (1229.70)	5,012.60 (1302.51)	−335 (-3,708 to 3,320)
Productivity loss^a^	4,002.20 (1484.43)	2,502.57 (1055.98)	−1,500 (-4,987 to 1,907)
*Total costs* ^a^	*13,477.71 (2450.28)*	*10,843.50 (1747.89)*	*−2,643 (-8,535 to 3,058)*

*GA* graded activity, *EXP* gradual exposure, *QBPDS* Quebec Back Pain Disability Score, *QALYs* quality-adjusted life year, *P & F costs* patient and family costs

^a^Difference in mean (95 % confidence interval) derived using bootstrapping (1,000 replications)

^b^Difference in mean (95 % confidence interval) derived using independent samples *T*-test

### Resource use utilization and productivity loss

During the one-year follow-up, EXP patients utilized fewer resources than GA patients, with the exception of prescribed medication, hours of unpaid help and unpaid work (see Table 3).

**Table 3:** Mean (SD) resource utilization per patient by treatment group

Type of utilization	Mean (SD)	Mean (SD)	
	GA (*N* = 31)	EXP (*N* = 31)	Mean difference (95 % CI)^b^
Number of contacts:			
General practitioner	2.74 (4.67)	2.13 (3.49)	−0.65 (-2.82 to 1.34)
Medical specialist	2.02 (3.25)	1.98 (2.84)	−0.02 (-1.50 to 1.50)
Other health care providers^a^	29.44 (51.01)	17.39 (27.10)	−12.44 (-32.53 to 5.87)
Alternative health care	2.58 (5.53)	1.94 (9.44)	−0.63 (-3.71 to 3.63)
Prescriptions for medication:			
Anxiolytics	0.24 (0.99)	0.69 (2.66)	0.46 (-0.40 to 1.53)
Antidepressants	0.16 (0.90)	0.32 (1.41)	0.13 (-0.40 to 0.73)
Opioids	1.29 (3.59)	0.65 (1.93)	−0.70 (-2.26 to 0.57)
Other medication	0.81(3.38)	1.17 (4.99)	0.38 (-1.61 to 2.58)
Over-the-counter medication	0.23 (0.50)	0.29 (0.53)	0.06 (-1.96 to 3.25)
Hospitalization (in days)	0.48 (2.69)	0	
Hours of help:			
Home care	45.24 (176.96)	10.32 (40.00)	−35.16 (-112.26 to 12.02)
Paid domestic work	5.16 (18.28)	10.32 (31.08)	3.43 (-7.26 to 17.07)
Unpaid help	168.23(348.08)	179.26 (321.13)	19.26 (-140.76 to 182.94)
Hours absent from:			
Unpaid work	308.99 (416.81)	332.92 (442.99)	14.44 (-188.02 to 228.64)
Usual activity	49.72 (100.98)	37.89 (76.05)	−12.08 (-58.31 to 31.26)
Paid work	291.69 (501.99)	191.39 (362.60)	−104.81 (-324.84 to 103.69)

*GA* Graded activity, *EXP* Exposure in vivo

^a^Physiotherapist, occupational therapist, manual therapist, Mensendieck therapist, Cesar therapist, psychologist, dietician, pedicurist, masseur, orthopedist, and social worker

^b^95 % confidence intervals derived with independent samples *T*-test

### Costs

The costs of the GA and EXP treatments were calculated by multiplying the number of sessions by the costs of the treatment team involved and travel expenses to the sessions. Although GA treatment involved more sessions (25 compared to 16), EXP treatment appeared to be more expensive due to the involvement of the psychologist in almost every session. Table 2 presents the mean health care costs (including the intervention costs), patient and family costs, and production losses of both interventions and the 95 % confidence intervals obtained by bootstrapping. All cost comparisons (except for intervention costs) between EXP and GA were in favor of EXP, which means that patients who received EXP treatment incurred fewer total costs in the post-treatment year compared to those who received GA treatment.

### Cost-effectiveness planes, CEACs and uncertainties

**Fig. 1:**
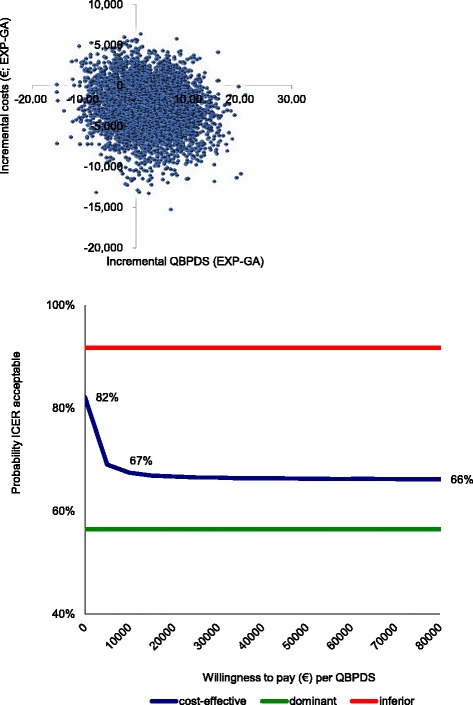
Cost-effectiveness plane and CEAC for QBPDS for EXP versus GA. EXP = exposure in vivo, GA = graded activity, CEAC = cost-effectiveness acceptability curve, QBPDS = Quebec Back Pain Disability Scale

The EXP program resulted in a greater mean health benefit (2.2 improvement on the QBPDS) achieved at a lower mean total cost (€2,634 cost savings) compared to GA. The cost-effectiveness plane for the QBPDS, representing the 5,000 bootstrap replications, is shown in Fig. 1. The dominance of EXP treatment (i.e., less disability at lower cost) was shown in 56 % of the replications (i.e., 56 % of bootstrapped cost-effect pairs were in the southeast quadrant). Of all bootstrapped likelihood replications, 8 % were located in the northwest quadrant (indicating inferiority), 26 % in the southwest quadrant (indicating that GA is cheaper but also results in less reduction of disability) and 10 % in the northeast quadrant (implying that EXP is more expensive but also more effective).

Figure 1 also presents the CEAC of the cost-effectiveness analysis based on the QBPDS. Given a €16,000 willingness to pay for an additional improvement on the QBPDS, the probability of EXP treatment being cost-effective is 67 %.

In addition, the ICER based on QALYs showed a greater health benefit (0.01 additional QALYs) and lower mean total cost (€2,643 cost savings) in favor of EXP treatment.

**Fig. 2:**
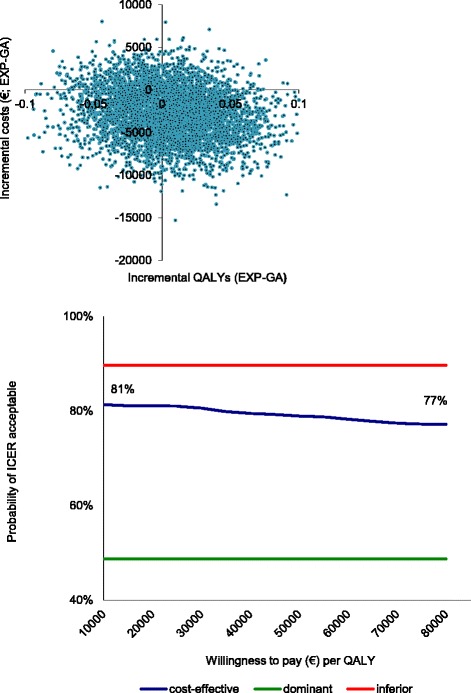
Cost-effectiveness plane and CEAC for QALYs for EXP versus GA. EXP = exposure in vivo, GA = graded activity, CEAC = cost-effectiveness acceptability curve, QALYs = quality-adjusted life years

Uncertainty analyses (shown in Fig. 2) were calculated using 5,000 bootstrap replications of the cost-utility ratio for QALYs comparing EXP to GA treatment. In this plane, the dominance of EXP was shown in 49 % of the replications. Only a small number (10 %) are located in the inferior quadrant, indicating the superiority of EXP. Figure 2 also presents the CEAC of the cost-utility analysis based on QALYs. Given a €16,000 willingness to pay for an additional QALY, the probability of EXP treatment being cost-effective is 81 %. When the maximum ceiling ratio of €80,000 is used, the probability diminishes slightly, to 76 %. This small decrease indicates that the final decision is rather insensitive to the ceiling ratio chosen.

In addition, one-way sensitivity analyses were performed using the main assumptions of the Friction Cost Approach instead of the Human Capital Approach. This reduced the productivity costs and, as a consequence, the total costs in both groups. Since only two patients (one in each group) were absent for more than 23 weeks, the reduction in costs was almost equal between the groups, which does not change the conclusion reached using the Human Capital Approach. Finally, since absenteeism accounts for more than 50 % of the total cost, we bootstrapped the ICERs of the CEA and the CUA once more, now excluding productivity costs fully. This leads to a decreased percentage dominant ICERS for the CEA and the CUA, resp. 51 % and 46 %.

## Discussion

This is the first economic evaluation study to compare the use of EXP and GA to treat chronic low back pain. In line with our hypothesis, economic analysis showed that EXP might be the more optimal treatment for CLBP patients with moderate to high fear of movement, injury or reinjury compared to GA treatment. An uncertainty analysis found that EXP is preferable to GA in terms of costs and utilities; this finding is robust and insensitive to the ceiling ratio chosen. EXP showed a tendency to improve functioning and QALYs and reduce total health care and productivity costs.

As the ultimate objective of an economic evaluation study is to find the difference in cost-effectiveness (incremental cost per effect/QALY), changes to the effects/QALYs only are not interesting without also considering the cost of bringing about such changes [43]. Both incremental cost-effectiveness ratios resulted in more health benefits (functioning and QALYs) and lower total costs for EXP. In addition, bootstrap simulations exploring sample uncertainties were also favorable for EXP. Depending on the threshold range, the probability of EXP being cost-effective compared to GA varies between 83 % (threshold €16,000/QALY) to 76 % per QALY (threshold €80,000/QALY). Thus, regardless of the threshold (which varied in this study between €0 and €80,000) [27], the final choice for EXP is rather robust and insensitive to the ceiling ratio chosen. Nevertheless, the probability of EXP being cost-effective is a bit less for functioning, varying between 81 % and 67 %. Although this is still large, one should take into account that this also means that 33 % of the patients might not benefit from EXP.

Looking at the cost-effectiveness results in greater detail, the cost per QALY (derived from the SF-6D utilities) were more favorable for EXP than GA when compared to the cost per self-reported disability. However, there were improvements compared with baseline for both interventions: the difference between baseline and follow-up SF-6D scores exceeded the proposed minimal important difference of 0.03 for this instrument [43, 44], which indicates that this seemingly small difference can be regarded as beneficial enough for patients and health care professionals. On the cost side, EXP treatment was more expensive when only considering the costs during the intervention. However, during the 12-month follow-up after the intervention, all the other health care and productivity costs were higher for the participants who completed the GA intervention, resulting in overall higher societal costs for GA.

As in earlier studies that looked at patients with non-specific chronic pain [7] or psychiatric problems [45], the societal costs consisted mainly of patient and family costs originating from out-of-pocket costs (e.g., for help needed at home, informal care, and additional expenses). In line with other studies looking at CLBP [6–9], this study revealed that productivity losses make up a large share of the societal costs. From an economic perspective, these results again underline the importance of applying a societal viewpoint and incorporating these costs in a cost-effectiveness analysis of patients with chronic pain. In addition, this supports the use of a bottom-up data source (e.g., in-depth questionnaires or patient diaries) instead of a top-down approach (e.g., using public or private health insurer claims databases or hospital databases) [9].

In our study, the data from the cost diaries gave us insight into the health care consumption of patients with CLBP during the 15-month follow-up. The patients in this study visited their general practitioners twice during this period, which was almost similar to the number of visits to an alternative care or medical specialist. By comparison, members of the general Dutch population visit their general practitioners 0.75 times and a medical specialist 0.4 times on average per year [46].

Visits to physical and occupational therapists were reported most often. Patients in the GA group visited a physical or occupational therapist almost twice as often as patients in the EXP group (29 versus 17 visits), which could be explained by the time-contingent treatment schedule with predefined end goals in the GA treatment. It is possible that GA patients were dissatisfied with their outcomes and continued to work with a physical or occupational therapist. Another possible explanation is that GA patients catastrophized more about the negative consequences of their pain and perceived harmfulness of activities [19] compared to EXP patients, and that this led to higher health care utilization.

Although not significantly different, the GA group reported more hours of absence from work in the year after the intervention than the EXP group did. We do not have a full explanation for this difference. Some of the hours could have been spent on physical/occupational therapist care, which could have occurred during working hours. The previously mentioned fear of pain and activities may be another explanation for the absenteeism. Although the productivity costs are a substantial part of the total cost, it has a relatively small impact on the bootstrapped ICERs.

Unlike the difference in costs between GA and EXP treatment (resulting in fewer societal costs for EXP), the difference in effects was negligible. This is in line with the earlier clinical study conducted by Leeuw et al. [19]. One possible explanation for this could be that the differences between the interventions were not as distinct as assumed. Some elements of EXP treatment may have been included in the GA interventions, such as fear-provoking activities and reassurance that all the activities were safe and allowed.

This study has some limitations. The cost-effectiveness and cost-utility study results were based on a small sample: only 62 respondents were included in the economic evaluation, instead of the requisite 110. Like almost all trial-based economic evaluations, this economic evaluation is powered on the main outcome of the effectiveness study. Though we accounted for sample uncertainty using bootstrapping, dropout may have been an issue. However, dropout was not related to the content of the treatment, and baseline characteristics of the patients included in the economic analyses did not differ from those excluded on account of a lack of economic data. Overall, the response rate was good, with 92 % completing the cost diaries. The bootstrap analysis, which corrected for the sample uncertainty, revealed that the study results are quite robust. Still, we believe that a larger sample might have resulted in significant results, and more certainty regarding costs and monetary benefits, which could have affected the result of the cost-effectiveness calculation.

Patient time was not included as a separate cost. According to the Dutch guideline, a patient’s time costs are equal to their productivity costs plus the impact of the illness-related change in spare time activities on their quality of life plus absenteeism. Spare time is not monetized since this could lead to double counting as it is included in the measurement of quality of life.

The generalizability of the results of economic evaluation studies has been discussed, which is linked to factors that can differ between places, such as the availability of health care resources, clinical practice patterns, prices, collection of resource use and cost data, and to study perspective and factors related to an RCT [47]. Our study was conducted alongside an RCT, which may have limited the generalizability. However, several aspects were taken into account to increase the transferability to other settings in the Netherlands and beyond. First, a broad societal perspective was adopted. This means that the level of decision-making is society as a whole. Second, the valuation of resource use data was based on the Dutch manual for cost analysis in health care research, using standardized cost prices [28]. Third, to enable other settings to apply their own prices to the unit of resource use, prices and resource use were documented separately. Fourth, the study was conducted at four treatment centers, thus better reflecting variation in health care provision. Fifth, and finally, health state preference values were derived from the generic SF-6D, which uses tariffs obtained from a community survey. This tariff is used as an international standard to derive utilities from the SF-36 [37]. The use of this generic outcome measure enables policymakers to make comparisons between different diseases.

Since this is the first economic evaluation study to compare EXP to GA treatment for CLBP, there are however no similar economic evaluation studies to allow comparison. A recent systematic review that investigated the results of cost-effectiveness studies of interdisciplinary rehabilitation and cognitive behavioral therapy concluded that these interventions were cost-effective for sub-acute and chronic low back pain, regardless of the comparisons and perspectives adopted [48]. Only one study has evaluated GA treatment in a population of people with CLBP [49], which revealed that a combination of GA treatment and problem-solving is more cost-effective than a combination of GA treatment with problem-solving and active physical treatment. To our knowledge, EXP treatment has never before been evaluated in an economic evaluation study.

## Conclusions

In conclusion, clinicians and policymakers should consider the use of EXP treatment for patients suffering from chronic low back pain since it seems to be more cost-effective than GA treatment. Nevertheless, since the study was underpowered, the conclusion should be treated carefully.

## Abbreviations

CBT: cognitive behavioral treatment
CEA: cost-effectiveness analysis
CEAC: cost-effectiveness acceptability curve
CLBP: chronic low back pain
CUA: cost-utility analysis
EXP: exposure in vivo
GA: graded activity
ICER: incremental cost-effectiveness ratio
PHODA: Photograph Series of Daily Activities
QALY: quality-adjusted life year
QBPDS: Quebec Back Pain Disability Scale
SD: standard deviation
SF-36: Short Form 36
SPSS: Statistical Package for Social Sciences
